# Acaricidal bioactivity and molecular target analysis of *Origanum onites* and *Ocimum gratissimum* essential oils against *Haemaphysalis doenitzi* ticks

**DOI:** 10.1186/s13071-025-07031-3

**Published:** 2025-10-08

**Authors:** Songbo Zhang, Zhihua Gao, Han Wang, Jingyao Gao, Feidi Guo, Runying Wang, Weijia Xing, Jianing Liu, Xinyu Zhang, Xiaolong Yang

**Affiliations:** Hebei Key Laboratory of Animal Physiology, Biochemistry and Molecular Biology, Hebei Collaborative Innovation Center for Eco-Environment, Ministry of Education Key Laboratory of Molecular and Cellular Biology, College of Life Sciences, Hebei Normal University, Shijiazhuang, 050024 China

**Keywords:** *Origanum onites*, Carvacrol, Acaricidal activity, Repellent activity, ATP-binding cassette transporter, Biochemical assay

## Abstract

**Background:**

*Haemaphysalis doenitzi* is a parasite mainly found on the body surface of birds that is capable of transmitting rickettsiae and borrelia, which can cause serious zoonotic diseases. Chemical acaricides are controversial because they pollute the environment and predispose ticks to resistance. In contrast, plant essential oils (EOs) are favored for their effective acaricide properties and environmental friendliness.

**Methods:**

The constituents of *Origanum onites* and *Ocimum gratissimum* EOs were profiled using gas chromatography-mass spectrometry (GC-MS). Acaricidal activities were evaluated by immersing unfed nymphs and adults of *H. doenitzi* in serial solutions for 5 min and monitoring mortality after 24 h. Enzyme activities (Na⁺/K⁺-ATPase, GST, CarE, AChE) and transcript levels of HDABCE1, HDCYP450a and HD-GSTa were quantified in homogenates of treated survivors. Homology models of the three target proteins were generated and docked with carvacrol and eugenol to predict binding sites and affinities.

**Results:**

GC–MS analysis showed 93.3% of carvacrol in *O. onites* EO and 66.68% of eugenol in *O. gratissimum* EO. Immersion test showed that *O. onites* EO had significant acaricidal activity against nymphs and adults, with median lethal concentration (LC_50_) values of 14.694 mg/ml and 24.357 mg/ml, respectively. *Ocimum gratissimum* EO showed significant acaricidal activity against nymphs and adults, with LC_50_ values of 9.379 mg/ml and 18.299 mg/ml, respectively. Carvacrol also showed significant acaricidal activity against unfed nymphs and adults. Repellency tests showed that *O. onites* EO had more significant repellent activity against nymphs than DEET, with median effective concentration (EC_50_) values of 2.162 mg/ml and 7.039 mg/ml, respectively. To explore the molecular mechanisms of *O. onites* EO and carvacrol on ticks, we investigated the enzyme activity and gene expression of ATP-binding cassette transporter, cytochrome P450, and glutathione S-transferase. Finally, molecular docking was used to verify the enzyme effects.

**Conclusions:**

The results of this study provide important insight into the toxicity mechanisms of ticks, and indicate that carvacrol and *O. onites* EO can be used as alternatives to chemically synthesized acaricides.

**Graphical Abstract:**

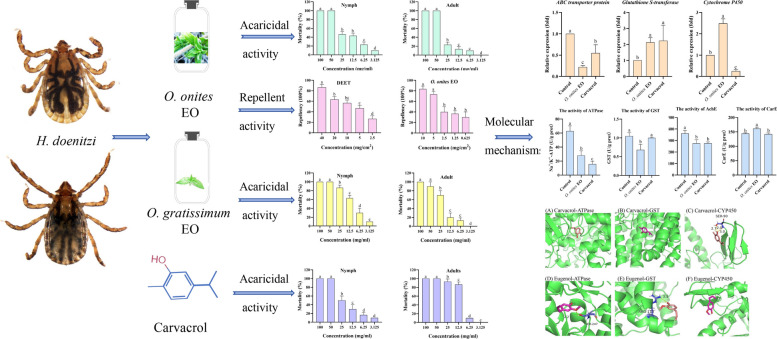

**Supplementary Information:**

The online version contains supplementary material available at 10.1186/s13071-025-07031-3.

## Background

Ticks, the second most important blood-feeding arthropod after mosquitoes, are involved in the transmission of a wide range of pathogens that pose a serious threat to human and animal health [1, 2]. Ticks also cause economic losses of up to billions of dollars to the global meat industry by parasitizing livestock such as free-range cattle and sheep [3]. *Haemaphysalis doenitzi* is a tick species in the Ixodidae family that can harm animals and humans [4, 5].It is widely distributed in Southeast Asia, including southern and northern China, India, Laos, Malaysia, Myanmar, the Philippines, Singapore, Sri Lanka, Thailand, and Vietnam [6]. It is widely distributed in various towns on Chongming Island, China, where it infests humans and predominantly parasitizes the body surfaces of small mammals [7]. It has been found that *H. doenitzi* is severely harmful to endangered wild birds [8, 9].

The ecological impact of ticks on the environment is often underestimated. Many species are affected, with wildlife such as birds, deer, and rabbits dying from bloodsucking [10, 11]. Chemical control is widely used in pest control because of its mature technology, large-scale production, rapid results, and market competitiveness [12]. However, ticks have gradually developed resistance to chemical acaricides due to long-term exposure. Therefore, the extraction and isolation of new active ingredients from plants has become a key pathway for the development of novel tick control agents [13, 14]. Oregano and basil belong to the Lamiaceae family and are widely used edible herbs worldwide for their excellent antioxidant and antimicrobial properties in food and medicine [15, 16]. Phenolic compounds from oregano also possess antioxidant and antiviral activity [17]. *Origanum onites* essential oil (EO) and its constituents have been found to have significant toxic effects on *Thaumetopoea wilkinsoni* larvae [18]. *Origanum onites* also has a significant repellency to the lone star tick [19]. Previous studies have found that *Ocimum gratissimum* and the main constituent, eugenol, have attraction and toxicity to *Apolygus lucorum* [20]. *Ocimum gratissimum* EO has a complex composition with synergistic effects between its main constituents. Therefore, the development of a wide range of plant EOs will not only be effective in controlling tick-borne pests, but will also provide a scientific basis for large-scale ecological conservation and contribute to a wider discussion on environmental protection [21].

Plant EOs are excellent pesticides and are not only effective in minimizing risks to livestock and human health but also have significant economic value from a crop production perspective [22, 23]. This study aimed to investigate the chemical composition, insecticidal potential, and repellency of EOs from the leaves of *O. onites* and *O. gratissimum*, using *H. doenitzi* as a tick pest model. In addition, ATP-binding cassette (ABC) transporter proteins and glutathione S-transferases (GST) promote toxin excretion, cytochrome P450 (CYP450) and carboxylesterases (CarE) oxidize and hydrolyze insecticides, and acetylcholinesterase (AchE) regulates neural signaling [24, 25]. These mechanisms fuel tick resistance to chemical acaricides, and a deeper understanding of them is essential for developing new control strategies. Molecular docking analysis of the major compounds in these two EOs with these key enzymes was also performed to reveal their binding modes. From an environmental conservation perspective, these research results provide strong support for the development of green and environmentally friendly tick control methods that can help reduce the use of chemical insecticides and pollution, while maintaining the balance and diversity of ecosystems.

## Methods

### Essential oils and tick rearing

The EOs were obtained from Ji’an Huaxin Natural Essential Oils Mall (https://jian016646.11467.com/). *Origanum onites* and *O. gratissimum* EOs are produced in southwestern China, and the EO is extracted from the leaves by steam distillation. The fresh leaves are cleaned and placed in a distillation pot and submerged in water. Heating produces vapors, and the mixture cools and flows into the receiver, where the EOs are layered with moisture. The oils are then separated with a dispensing funnel, collected and stored in a dark glass bottle, and sealed and kept from light and heat. This extraction method is low-cost, simple, and easy to optimize. The equipment is simple and does not require complex chemical treatment, which reduces production costs. Optimizing the distillation temperature and time also improves extraction efficiency and EO quality. Carvacrol (99%; batch number C804847), a solid, was purchased from Shanghai MACKLIN Biochemical Technology Co., Ltd. (Shanghai, China). DEET mosquito repellent (99.3%) was purchased from Wuhan Weishi Bo Environmental Protection Technology Co.

Adequate numbers of adult ticks were collected from sheep in Cangxi County, Sichuan Province, and brought back to the laboratory. Ticks were identified using a Motic SMZ-140 series stereomicroscope (Xiamen, China). Ticks were placed in cloth bags over rabbit ears for blood-feeding, following the Animal Ethical Code (approval number 2023LLSC040). Engorged female ticks were kept in an incubator at 26 ± 1 °C with a 6 h light/18 h dark cycle and humidity above 80% to lay eggs. After hatching, larvae were fed on rabbits for 3 to 5 days, then incubated for 12–15 days to molt into nymphs. Nymphs were fed again for 3–6 days, collected, and incubated for 13–17 days to molt into adults. These nymphs and adults were used for experiments.

### Determination of the EO composition by gas chromatography–mass spectrometry (GC–MS)

GC–MS analysis was performed using an Agilent 5975 C gas spectrometer with an Agilent HP-5 capillary column (30 m × 0.25 mm × 0.25 μm film thickness). Helium was used as the carrier gas at a flow rate of 1 ml/min. The split ratio was 10:1, and the injection volume was 1.0 μl. The injector temperature was 250 °C. The heating program was as follows: 80 °C for 2 min, then 150 °C at 4 °C/min, 200 °C at 3 °C/min, and finally 260 °C at 10 °C/min for 15 min.

The mass spectrometry conditions included electron ionization (EI) as the ionization mode, electron energy of 70 eV, ion source temperature of 230 °C, interface temperature of 260 °C, and quadrupole temperature of 150 °C. Tuning was standard, scanning was a full scan, and the quality range was 50–550 amu. *Origanum onites* EO was diluted to specific concentrations and analyzed under the same experimental conditions, recording the retention time of each peak. The major constituents of the EOs were determined by comparing the mass spectra and retention indices (RI) with data from the National Institute of Standards and Technology (NIST) database or data provided in the literature.

### Nymph and adult immersion test

The acaricidal activity of EOs and carvacrol was assessed by nymph and adult immersion tests [26, 27]. The two EOs and carvacrol were tested at six concentration gradients of 100, 50, 25, 12.5, 6.25, and 3.125 mg/ml. The procedure was as follows: First, 10 unfed nymphs or 10 unfed adults were immersed in various concentrations of *O. onites* EO or carvacrol solution for 5 min, then dried on filter paper, and incubated for 24 h in an incubator. At the end of the incubation, the number of tick deaths was observed and counted using a stereoscopic microscope. Mortality was subsequently calculated for unfed nymphs and adults. Experiments were performed in triplicate for each treatment. To configure 2% Tween 80 solvent, 2 ml of Tween 80 and 98 ml of doubly distilled water (ddH_2_O) were used; the treatment group was doubly diluted with 2% Tween 80. The control group was 2% Tween 80.

### Nymph repellency tests

The repellency of *O. onites* EO and DEET was assessed by nymph repellency tests [28, 29]. The DEET was tested at five concentration gradients of 40, 20, 10, 5, and 2.5 mg/cm^2^. The *O. onites* EO was tested at five concentration gradients of 10, 5, 2.5, 1.25, and 0.625 mg/cm^2^. Whatman no. 4 filter paper was cut into a 7 × 4 cm rectangle, and a 1 × 4 cm horizontal line was drawn at each end; these areas were the untreated area, and the center 5 × 4 cm was the experimental area. Then, 165 µl 2% Tween 80 of the plant EO solution was dropped on the experimental area, allowing it to spread evenly, following by drying for 3 min. Ten unfed ticks were placed in the untreated area at the lower end of the filter paper. The position of the tick was observed after 5 min. If the tick remained in the untreated area or fell off without crossing the experimental area, the drug was considered to have an avoidance effect. The test was repeated three times. Repellency (%) = number of repellent/totals × 100%.

### Gene cloning

The ABC transporter detoxification gene from ticks was cloned. This gene is a candidate for metabolic resistance in ticks, and its expression levels indicate whether reagents are involved in tick resistance detoxification mechanisms. Primers for this gene were designed using Primer 5.0 software (forward primer: 5′-ATGAGTTCACAGGACAAGCTAACG-3′; reverse primer: 5′-TCAATCTTCAAGGAAAAAGAAGTTTC-3′) and used for polymerase chain reaction (PCR) amplification.

The median lethal concentration (LC_50_) of *O. onites* EO and carvacrol was used as treatment, and 2% Tween 80 was used as solvent control. DNA was extracted from adult ticks that survived the 24 h immersion test. The PCR reaction conditions were as follows: 94 °C for 5 min, followed by 30 cycles of 94 °C for 30 s, 59.5 °C for 30 s, and 72 °C for 1 min, with a final extension at 72 °C for 10 min. The PCR products were detected by 1% agarose gel electrophoresis and sequenced. The sequence was deposited in GenBank (National Center for Biotechnology Information [NCBI]) under accession number XOD50192. The ABC transporter protein sequence was aligned with homologous sequences in GenBank and analyzed for physicochemical parameters, and a phylogenetic tree was constructed using the maximum likelihood method and MEGA11 software.

### RNA extraction and quantitative real-time PCR (qPCR)

In this experiment, 30 unfed adult ticks were treated with LC_50_ concentrations of *O. onites* EO and carvacrol. After 24 h, surviving ticks were frozen in liquid nitrogen. RNA was extracted using the TransZol Up Plus RNA kit, and complementary DNA (cDNA) was synthesized from 4 μg of RNA using the TransScript™ one-step genomic DNA (gDNA) removal and cDNA synthesis kit. The cDNA was then diluted 10-fold and stored at −20 °C.

Primers for the ABC transporter gene were designed based on conserved regions. The GST gene (*HD-GSTa*) and CYP450 gene (*HD-CYP450a*) from *H. doenitzi* were cloned previously, and qPCR primers were designed based on these studies [5]. The primers were used to amplify cDNA by PCR, and the products were visualized on 1.5% agarose gel. After sequencing, the β-actin gene was chosen as an internal control. qPCR was performed using TranScript^®^ Green qPCR SuperMix (TransGen Biotech) with the following conditions: 45 °C for 5 min, 94 °C for 30 s, followed by 45 cycles of 94 °C for 5 s, 60 °C for 15 s, and 72 °C for 10 s. The relative expression levels of *HDABCE1*, *HD-GSTa*, and *HD-CYP450a* genes were determined using the 2^−ΔΔCt^ method (Table S1).

### Enzyme assays

The virulence toxin response of *H. doenitzi* was studied using ATPase and GST enzymes. Unfed adults (20 per group) were treated with LC_50_ concentrations of *O. onites* EO and carvacrol after 24 h, then immersed in liquid nitrogen. Frozen ticks were placed in 1.5 ml pre-cooled centrifuge tubes, and 300 μl of pre-cooled phosphate buffer (PBS, pH 7.4, containing 1 mM phenylmethylsulfonyl fluoride [PMSF]) was added, followed by homogenization with 2 mm-diameter stainless-steel beads at −4 °C for 180 s at 30 Hz. After centrifugation at 10,000×*g* for 10 min at 4 °C, the supernatant was filtered and placed on ice. Each group weighed 20 mg, and each treatment was repeated three times; 2% Tween 80 was used as a control. ATPase activity was measured using a Na^+^/K^+^-ATPase enzyme activity assay kit (Solarbio, BC0065). The enzyme activity was determined by measuring inorganic phosphorus production, with one unit defined as the amount of Na^+^/K^+^-ATPase that hydrolyzes ATP to produce 1 μM of inorganic phosphorus per gram of tissue per hour. Absorbance values were measured at OD [optical density] 660 after 10 min, and the activity was calculated as follows: Na^+^/K^+^-ATPase (U/g prot) = 7.5 × (A treated group − A control group) / (A standard group − A blank group) / Sample mass × Dilution ratio. GST activity was measured using a GST activity assay kit (Solarbio, BC0355). AchE activity of homogenates (20 μl) was determined using an AchE assay kit (Solarbio, Beijing, China, BC2025), and CarE activity of homogenates (10 μl) was measured using a CarE assay kit (Solarbio, Beijing, China, BC0845). GST, CarE, and AchE enzyme activity was calculated based on methods described in previous studies [11].

### Molecular docking

To study the interaction of carvacrol and eugenol with ATP transporters (ATPase), GST, and CYP450, we used the SWISS-MODEL website for protein homology modeling and obtained the three-dimensional structure of carvacrol and eugenol from the PubChem database. Molecular docking simulations were performed using AutoDockTools 1.5.7, and ligand–protein binding results were presented using PyMOL software.

### Statistical analyses

The data were analyzed using PoloPlus 1.0, IBM SPSS Statistics 24.0, JMP 14, and GraphPad Prism 8.0.2 software. Specific methods included analysis of variance (ANOVA). In addition, the regression equations were calculated in Excel. The level of significance was set at *P* < 0.05.

## Results

### EO constituents

The EO of *O. onites* was intensively analyzed for its chemical composition by the GC–MS technique, and four major compounds were identified along with their relative content (Table 1). The results showed that carvacrol was significantly dominant among these compounds, with relative content of 93.3%, and was the major component in the EO. It was followed by limonene, with relative content of 4.26%. 2-(1,1-Dimethyl ethyl) had relative content of 2.16%, whereas 5-methyl-2,4-diisopropylphenol had the lowest content of 0.1%. For *O. gratissimum* EO, GC–MS also showed relative percentages of six main compounds (Table 2). Among them, eugenol ranked first, with relative content of 66.68%. Caryophyllene was the second-largest constituent, with relative content of 13.94%. 1,4,7-Cycloundecatriene,1,5,9,9-tetramethyl-,Z,Z,Z had relative content of 4.31%. The relative content of naphthalene,1,2,3,5,6,8a-hexahydro-4,7-dimethyl-1-(1-methylethyl)-,(1S-cis) was 1.10%, and the relative content of caryophyllene oxide was 1.14%. The relative content of 2-butenedioic acid(E)-,bis(2-ethylhexyl) ester was 4.50%.

**Table 1:** Four main chemical compositions of *O. onites* oil

EO	Extract part	No.	RT (min)	Constituent	RIa	RIb	Area (%)
*O. onites*	Dry leaf	1	5.19	Limonene	1050	1048	4.26
		2	12.53	Carvacrol	1262	1262	93.3
		3	19	Phenol,2-(1,1-dimethyl ethyl)	1554	1557	2.16
		4	58.58	5-Methyl-2,4-diisopropylphenol	1512	1510	0.1
		Total					99.82

*RT* retention time, *RIa* retention index calculated by linear interpolation relative to retention times of a standard mixture of n-alkanes (C7–C40) using an HP-5MS column, *RIb* retention index from literature. Area (%): percentage of the chemical compound

**Table 2:** Six main chemical compositions of *O. gratissimum* oil

EO	Extract part	No.	RT (min)	Constituent	RIa	RIb	Area (%)
*O. gratissimum*	Dry leaf	1	8.059	Eugenol	1388	1392	66.68
		2	8.307	Caryophyllene	1453	1446	13.94
		3	8.450	1,4,7,-Cycloundecatriene,1,5,9,9-tetramethyl-,Z,Z,Z	1527	1519	4.31
		4	8.653	Naphthalene,1,2,3,5,6,8a-hexahydro-4,7-dimethyl-1-(1-methylethyl)-,(1S-cis)	1523	1520	1.10
		5	8.998	Caryophyllene oxide	1498	1507	1.14
		6	11.962	2-Butenedioic acid(E)-,bis(2-ethylhexyl) ester	2262	2224	4.50
		Total					91.67

*RT* retention time, *RIa* retention index calculated by linear interpolation relative to retention times of a standard mixture of *n*-alkanes (C7–C40) using an HP-5MS column, *RIb* retention index from literature. Area (%): percentage of the chemical compound

### Nymph and adult immersion test

The acaricidal activity of *O. onites* EO, *O. gratissimum* EO, and carvacrol was characterized against *H. doenitzi*, both nymphs and adults (Table 3). The results showed that *O. onites* EO had significant acaricidal activity against *H. doenitzi* nymphs and adults, with LC_50_ values of 14.694 mg/ml and 24.357 mg/ml, and 90% lethal concentration (LC_90_) values of 51.668 mg/ml and 57.052 mg/ml, respectively. *Ocimum gratissimum* EO had significant acaricidal activity against *H. doenitzi* nymphs and adults, with LC_50_ values of 9.379 mg/ml and 18.299 mg/ml, and LC_90_ values of 25.710 mg/ml and 47.164 mg/ml, respectively. Carvacrol showed significant acaricidal activity against *H. doenitzi* nymphs and adults with LC_50_ values of 16.509 mg/ml and 9.690 mg/ml, and LC_90_ values of 51.540 mg/ml and 17.053 mg/ml, respectively.

**Table 3:** Toxicity of essential oils from *O. onites* and *O. gratissimum* EOs against *H. doenitzi* nymphs and adults

Stage	Treatment	*n*	*df*	Regression eq. (Y =)	LC_50_ (95% CI)	LC_90_ (95% CI)	*χ*^2^	Slope ± SE
Unfed nymphs	*O. onites*	180	5	0.0091x + 0.2391	14.694^a^ (11.550–18.556)	51.668^b^ (37.460–84.386)	87.843	2.347 ± 0.307
	*O. gratissimum*	180	5	0.0078x + 0.393	9.379^a^ (7.457–11.535)	25.710^b^ (19.723–38.654)	94.579	2.926 ± 0.411
	Carvacrol	180	5	0.0097x + 0.1912	16.509^a^ (12.943–21.099)	51.540^b^ (37.257–85.512)	97.292	2.592 ± 0.326
Unfed adults	*O. onites*	180	5	0.0112x + 0.0425	24.357^b^ (18.669–32.611)	57.052^c^ (40.778–104.481)	132.361	3.467 ± 0.442
	*O. gratissimum*	180	5	0.0101x + 0.1566	18.299^b^ (15.006–22.314)	47.164^c^ (36.477–68.985)	110.899	3.117 ± 0.393
	Carvacrol	180	5	0.0083x + 0.3786	9.690^a^ (8.228–11.289)	17.053^b^ (14.244–22.605)	144.689	2.592 ± 0.326

*LC* lethal concentration (mg/ml), *CI* confidence interval, *df* degree of freedom. In each stage, the data followed by different letters of each column are significantly different at *P* < 0.05; *χ*^2^ = chi-square value; SE standard error

**Fig. 1:**
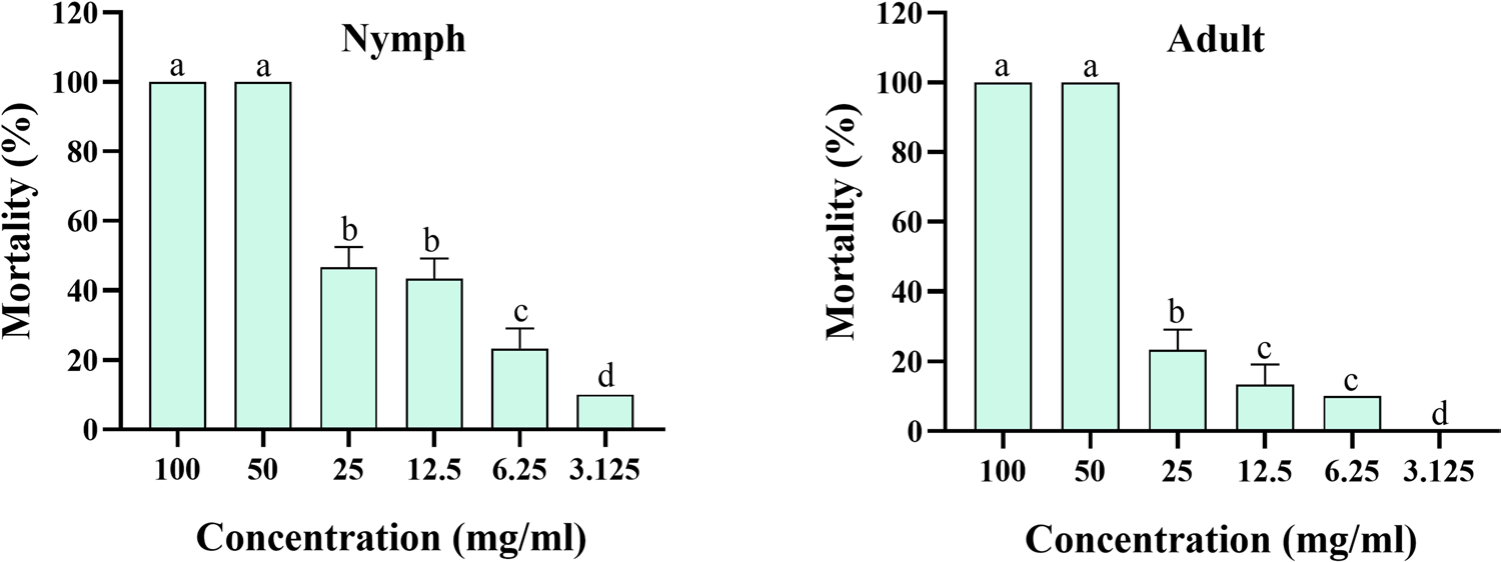
Mortality of *O. onites* EO against unfed nymph and adult *H. doenitzi* (columns with different letters are significantly different at *P* < 0.05).

**Fig. 2:**
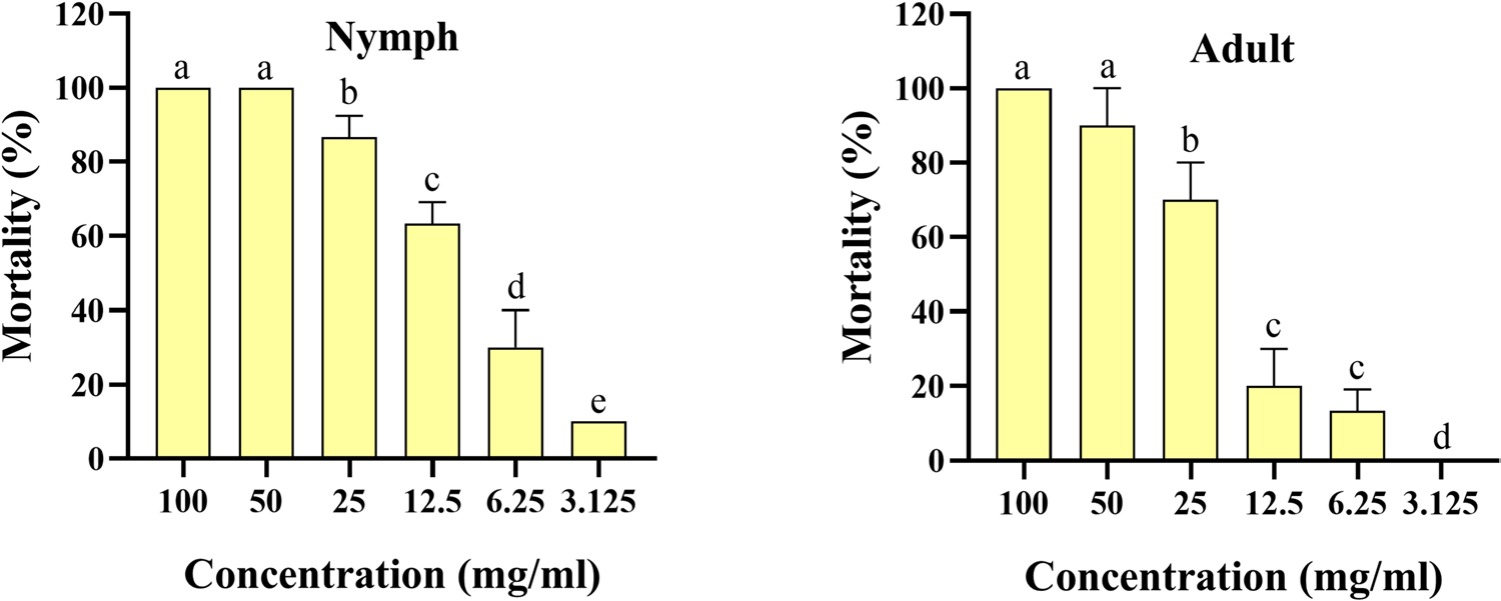
Mortality of *O. gratissimum* EO against unfed nymph and adult *H. doenitzi* (columns with different letters are significantly different at *P* < 0.05).

**Fig. 3:**
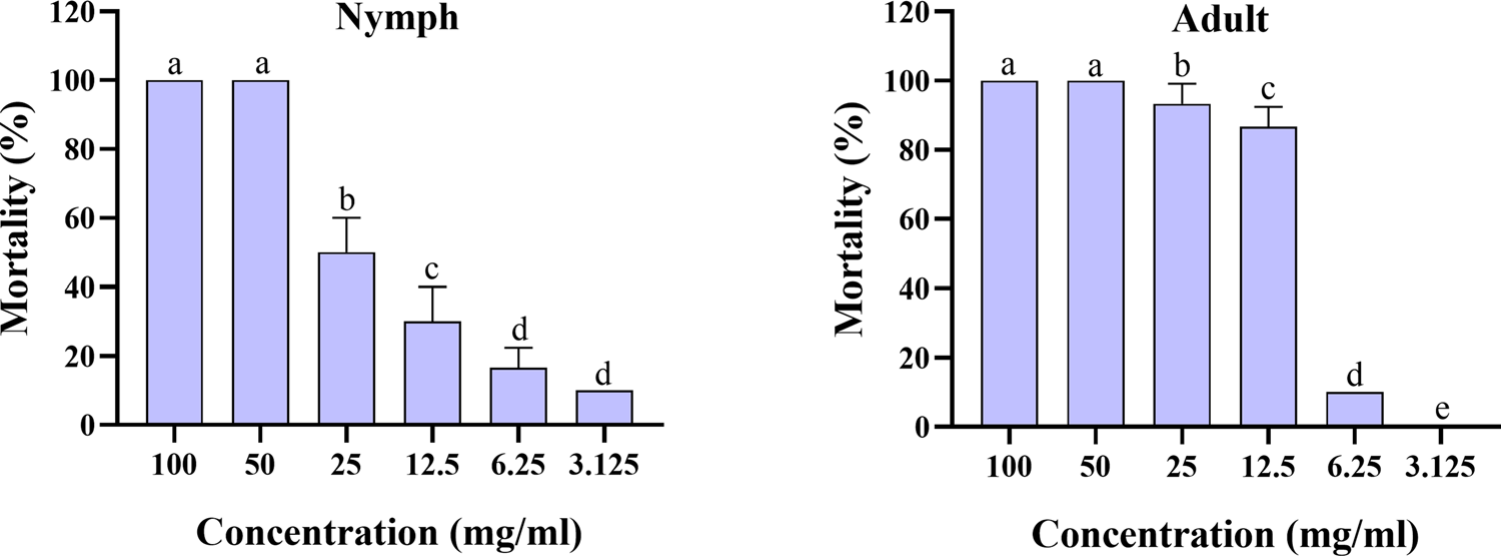
Mortality of carvacrol against unfed nymph and adult *H. doenitzi* (columns with different letters are significantly different at *P* < 0.05).

The mortality of nymphs and adults gradually increased with increasing concentrations of EOs and carvacrol (Figs. 1, 2, 3). *Origanum onites* EO was less toxic to unfed nymphs than to *O. gratissimum* EO (*P* < 0.05; Table 3). *Origanum onites* EO was less toxic than carvacrol to unfed adults *H. doenitzi* (*P* < 0.05).

### Repellency toxicity

**Fig. 4:**
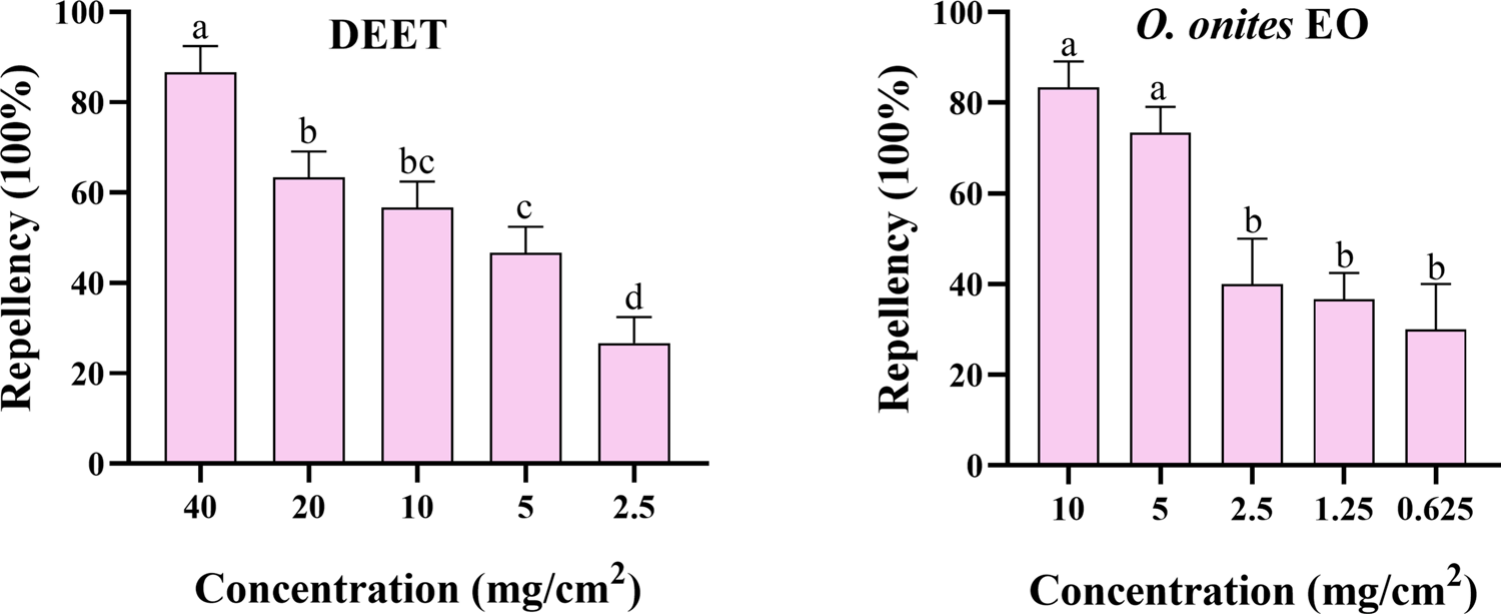
Repellency of *O. onites* EO against unfed nymph and adult *H. doenitzi* (columns with different letters are significantly different at *P* < 0.05).

The median effective concentration (EC_50_) values of *O. onites* EO and DEET were 2.162 mg/cm^2^ and 2.46 mg/cm^2^, and 90% effective concentration (EC_90_) values were 2.162 mg/cm^2^ and 2.46 mg/cm^2^ in the repellent tests with the unfed nymphs, respectively (Table 4). The above EC_50_ and EC_90_ values were calculated as the repellency rate of ticks 5 min after exposure. In the repellency test, both DEET at 40 mg/cm^2^ and *O. onites* EO at 10 mg/cm^2^ resulted in more than 80% repellency of nymph ticks. The *O. onites* EO repellency effect was significantly higher than DEET. The repellency of nymphs gradually increased with increasing concentrations of *O. onites* EO and DEET (Fig. 4).

**Table 4:** Repellent activity of essential oils from *O. onites* EO against unfed *H. doenitzi* nymphs

Stage	Treatment	*n*	*df*	Regression eq. (*Y* =)	EC_50_ (95% CI)	EC_90_ (95% CI)	*χ*^2^	Slope ± SE
Unfed nymphs	*O. onites*	150	4	0.0592*x* + 0.2972	2.162^a^ (1.587–2.888)	20.928^c^ (11.934–56.600)	276.520	1.300 ± 0.084
	DEET	150	4	0.041*x* + 0.45	7.039^b^ (5.020–9.328)	73.405^d^ (42.846–189.829)	236.472	1.259 ± 0.084

*EC* effective concentration (mg/cm^2^), *CI* confidence interval, *df* degrees of freedom. In each stage, the data followed by different letters of each column are significantly different at *P* < 0.05; *χ*^2^ = Chi-square value

### Gene cloning and sequence identification of *HDABCE1*

**Fig. 5:**
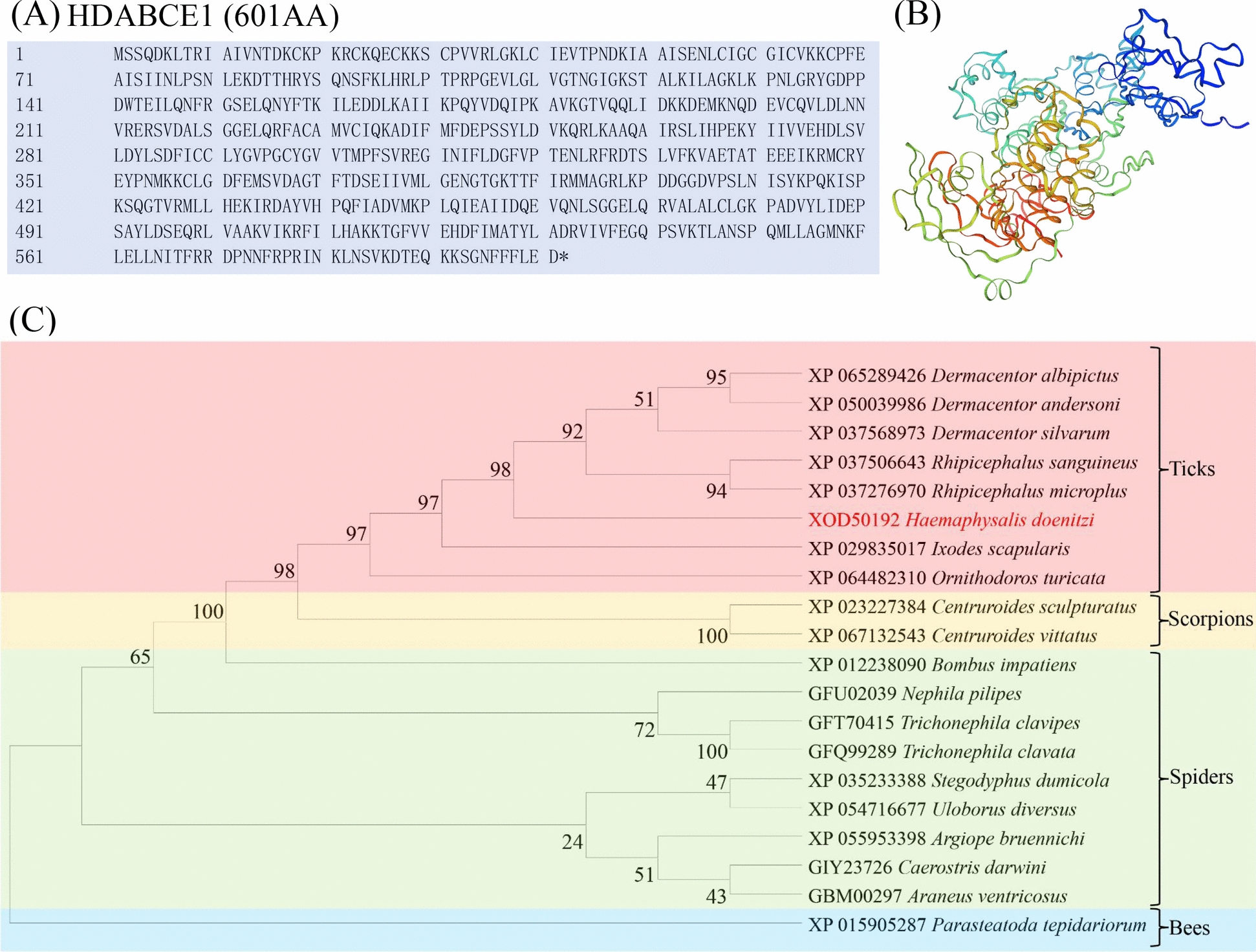
**A** The amino acid sequences of *HDABCE1* in *H. doenitzi*; **B** the predicted tertiary structures of *HDABCE1*; **C** phylogenetic tree of *HDABCE1* and their homologous ATP-binding cassette transporter protein subfamily E

The successful clone of the ABC transporter gene was named *HDABCE1*. The cDNA sequence of *HDABCE1* contains an open reading frame (ORF) of 1806 base pairs, which may encode 601 amino acids and contains Fe–S and AAA+ ATPase domains (Fig. 5A). The sequence is stored in the NCBI database under accession number XOD50192.1. *HDABCE1* has a predicted molecular weight of 67.59 kDa and an isoelectric point of 8.28. No signal peptide was predicted in *HDABCE1*, suggesting that it is a cytoplasmic ABC transporter protein. The predicted tertiary structures were calculated using the SWISS-MODEL (Fig. 5B). Phylogenetic analysis showed that *HDABCE1* clustered with ABC subfamily E member 1 genes in different species, and the results confirmed the high homology of family E in ABC (Fig. 5C).

### Expression profiles of *HDABCE1*, *HD-GSTa*, and *HD-CYP450a* genes

**Fig. 6:**
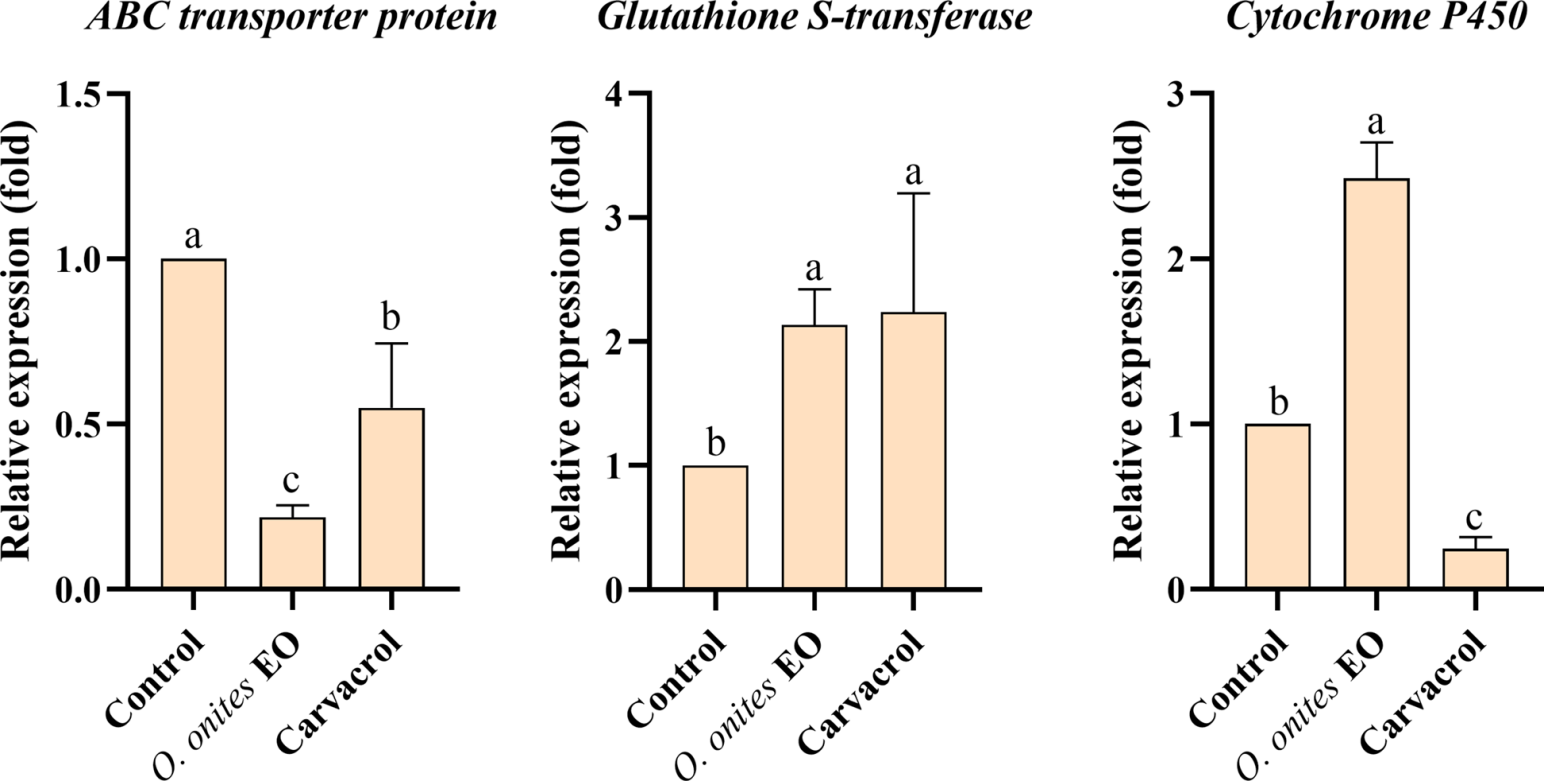
Effects of *O. onites* EO and carvacrol on *HDABCE1* (ABC transporter), *HD-GSTa* (glutathione S-transferase), and *HD-CYP450a* (cytochrome P450) expression in unfed adult *H. doenitzi* (columns with different letters are significantly different at *P* < 0.05).

The relative expression levels of three related detoxification enzyme genes in ticks treated with *O. onites* EO (LC_50_ = 24.357 mg/ml) and carvacrol (LC_50_ = 9.690 mg/ml) for 24 h were determined as follows (Fig. 6). The results of qPCR analysis showed a significant decrease in the expression of the *HDABCE1* gene in *O. onites* EO- and carvacrol-treated adults compared to the control treatment (2% Tween 80). The *HD-GSTa* gene was highly expressed in *O. onites* EO- and carvacrol-treated adults, with relative expression of 2.13 and 2.24-fold higher than that of the control. The inconsistent expression levels of the *HD-CYP450a* gene in ticks after treatment with *O. onites* EO and carvacrol may be attributed to the complex composition of the EO, which could involve antagonistic interactions among its components.

### Enzyme activity

**Fig. 7:**
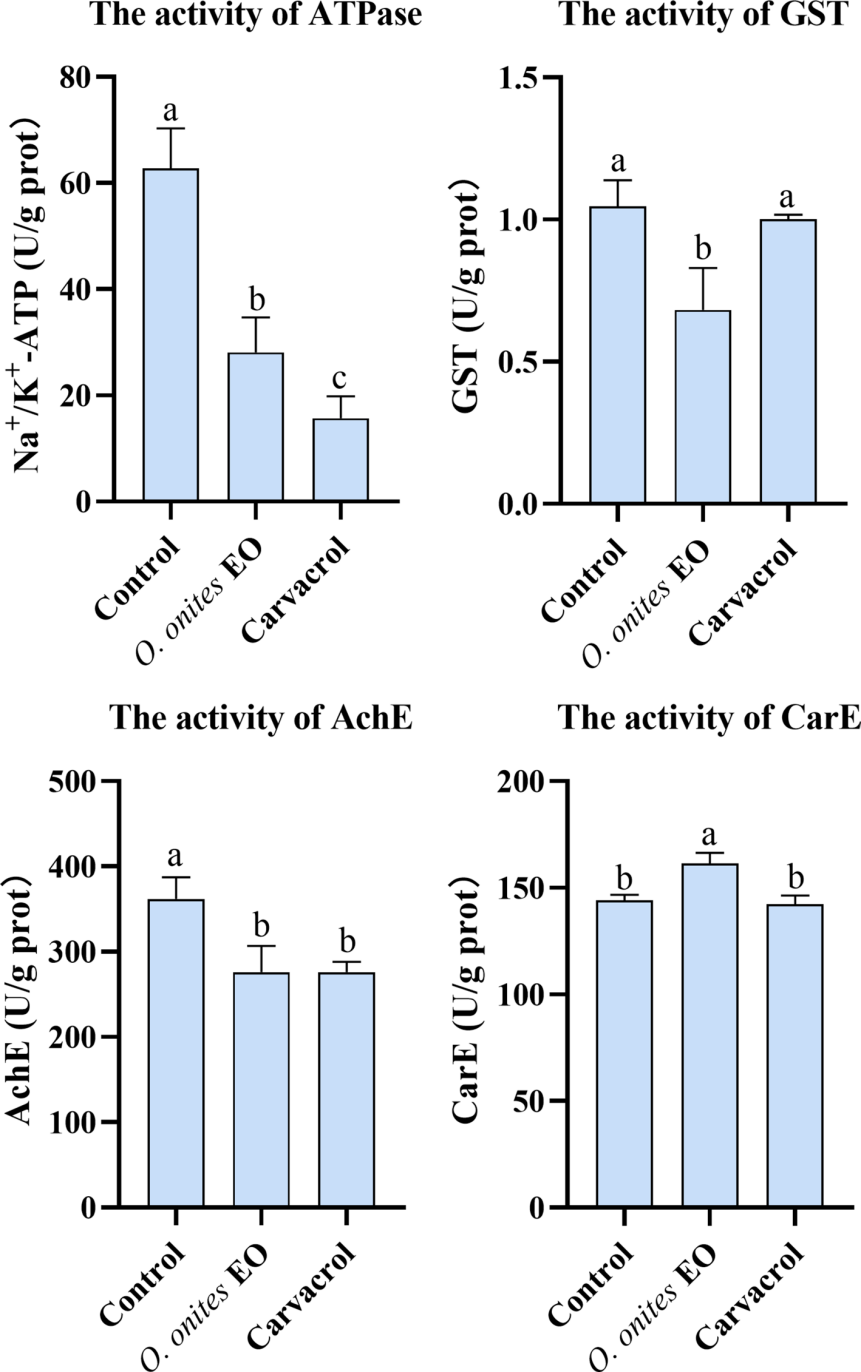
Effects of *O. onites* EO and carvacrol on ATPase, GST, CarE, and AchE of unfed adult *H. doenitzi* (columns with different letters are significantly different at *P* < 0.05)

The changes in Na^+^/K^+^-ATPase, GST, AchE, and CarE activity were characterized after treatment of adults with *O. onites* EO and carvacrol at LC_50_ (Fig. 7). The results showed that *O. onites* EO and carvacrol significantly inhibited ATPase activity (*P* < 0.05). The *O. onites* EO significantly inhibited the activity of GST (*P* < 0.05), but carvacrol had no significant effect on the activity of GST. *Origanum onites* EO and carvacrol significantly inhibited AchE activity (*P* < 0.05). *Origanum onites* EO significantly increased CarE activity (*P* < 0.05), whereas carvacrol had no significant effect on CarE activity. The differential effects of EO and carvacrol on GST and CarE activity may be influenced by other components in EO. The results showed that *O. onites* EO could affect the normal physiological and biochemical metabolism of ticks more significantly than carvacrol, leading to changes in the activity of different enzymes.

### Molecular docking

**Fig. 8:**
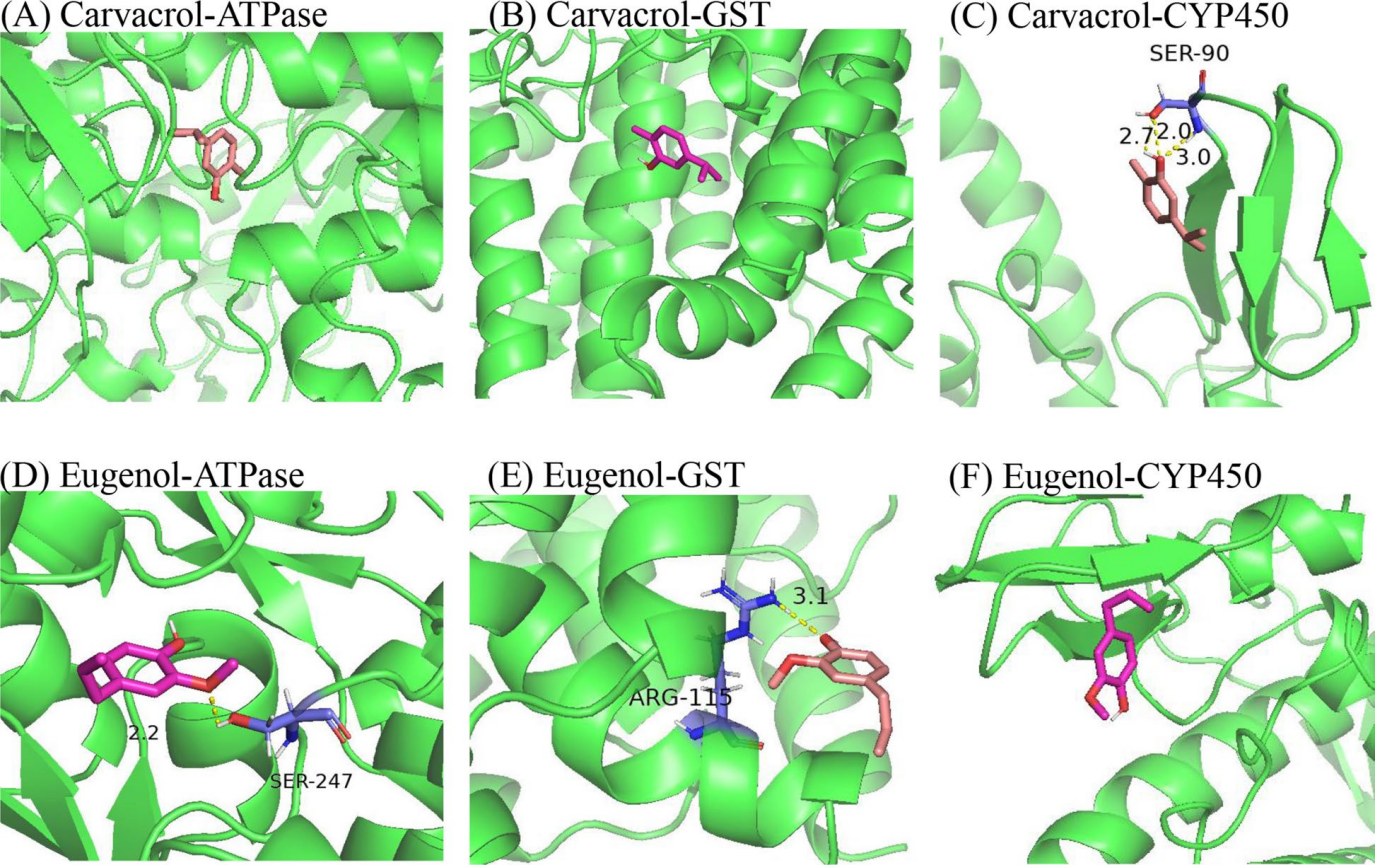
Selected complexes between molecules and EO components.** A**: Carvacrol-ATPase; **B**: Carvacrol-GST; **C**: Carvacrol-CYP450; **D**: Eugenol-ATPase; **E**: Eugenol-GST; **F**: Eugenol-CYP450

The results of molecular docking experiments (Table 5) showed that the binding energies of carvacrol with ATPase, CYP450, and GST were −3.65 kcal/mol, −2.66 kcal/mol, and −2.45 kcal/mol, respectively. The binding energies of eugenol with ATPase, CYP450, and GST were −2.15 kcal/mol, −1.59 kcal/mol, and −1.57 kcal/mol, respectively. Carvacrol showed the strongest binding affinity for ATPase, with the lowest value of all interactions tested. This finding aligns with the enzyme activity assays and qPCR results. The visualization results in Fig. 8 show that carvacrol forms three hydrogen bonds with Ser-90 of CYP450, which indicates strong binding, while eugenol forms one hydrogen bond with Ser-247 of ATPase and one hydrogen bond with Arg-115 of GST, which also indicates stable binding. Due to their molecular structures, carvacrol and eugenol cannot form hydrogen bonds with tick ATPase and GST, nor can eugenol form hydrogen bonds with tick CYP450. However, both compounds occupy the active sites of these enzymes through a combination of hydrogen bonds and hydrophobic interactions, thereby exerting their effects. Overall, the two compounds showed good binding ability to the three target proteins.

**Table 5:** Affinity of carvacrol and eugenol docking with three receptor protein molecules

Molecules	Carvacrol affinity (kcal/mol)	Eugenol affinity (kcal/mol)
ATPase	−3.65	−2.15
CYP450	−2.66	−1.59
GST	−2.45	−1.57

## Discussion

Plant EOs, as a new type of tick repellent and acaricide, can reduce environmental pollution and the problem of tick resistance, but their field of application is still limited by challenges such as compositional variability, high volatility, high dosage requirements, and environmental instability, so in-depth studies are needed [30, 31]. This study integrates EOs, key genes, and enzymatic indicators, which provides a new perspective for elucidating the overall detoxification mechanism of ticks to plant-derived toxicants, and establishes the theoretical and target framework for the development of green acaricides and delayed resistance.

Plant EOs have production potential and human health benefits due to their biological activity and pharmacological effects, and have significant acaricidal activity against ticks [32]. We examined the main constituents of *O. onites* and *O. gratissimum* EOs. The main component of *O. onites* EO is carvacrol, constituting 93.3%. In *O. gratissimum* EO, the primary constituents are eugenol (66.68%) and caryophyllene (13.94%). The EO of *O. gratissimum* has a complex composition, but the main component is eugenol, which can be mass-produced at low cost by hydrodistillation [33]. This study evaluated the acaricidal activity of *O. onites* EO, *O. gratissimum* EO, and carvacrol against nymph and adult ticks. Previous studies have also shown that *O. gratissimum* EO has significant acaricidal activity against *Rhipicephalus microplus* [34]. The lethal effect of *O. gratissimum* EO on unfed ticks was significantly better than that of *O. onites* EO and carvacrol. The acaricidal activity of *O. onites* EO against *H. doenitzi* is reported here for the first time. The lethal effect of *O. onites* EO on unfed nymphs was significantly better than that on adults. Curiously, the lethal effect of carvacrol on unfed adults was significantly better than that on the nymphs, probably due to physiological differences between nymphs and adults.

In a previous study, *O. onites*, *Origanum majorana*, and *Origanum minutiflorum* exhibited good acaricidal and repellent effects against *Rhipicephalus annulatus* larvae, killing 50% of tick larvae (LC_50_) at doses of 22.99, 25.08, and 27.06 μl/ml, respectively, with *O. onites* EO showing the highest repellent effect [35]. Similarly, this experiment confirmed that *O. onites* and DEET have equally potent repellent activity. The EC_50_ values of *O. onites* EO and DEET were 2.162 mg/cm^2^ and 2.46 mg/cm^2^, respectively. Although *O. gratissimum* is attractive to insects, it is a complex oil with comparable acaricidal activity to *O. onites*. In contrast, *O. onites* EO is superior in many respects: lower cost, easier extraction of key components, significant acaricidal activity, and repellency. Therefore, *O. onites* EO is not only more valuable but also a more efficient use of plant resources. Based on these advantages, we carried out an in-depth study of *O. onites* EO. Carvacrol has been shown to significantly interfere with the olfactory perception process of insects and to have a repellent effect [36]. The LC_50_ of carvacrol for unfed adults was 9.690 mg/ml, which was higher than the LC_50_ of *O. onites* EO for unfed adults of 16.509 mg/mL. This may be due to the interaction between carvacrol and other components in the EO, and there may be a mutual inhibitory effect [37]. Carvacrol, as a natural insecticide of plant origin, has antibacterial, antioxidant, anti-inflammatory, and other biological activity [38, 39]. Its acaricidal efficacy has been validated in a variety of studies, and it especially excels in tick control [40, 41]. Therefore, the present study provides an in-depth investigation of the mechanism of acaricidal activity of *O. onites* EO and carvacrol against ticks.

In this paper, the LC_50_ values for *O. onites* EO and carvacrol were selected for study to provide insights into the acaricidal activity of these two substances on adult *H. doenitzi* and their potential mechanisms. Concentration is a key factor in the use of EOs and their major components for tick control, as high concentrations can be harmful to non-target organisms [42, 43]. Understanding the toxin mechanism when the LC_50_ is applied can help to develop more economical and environmentally friendly plant-based acaricides, while the LC_50_ serves as a standardized index to ensure the comparability of results from different studies and facilitates the integration and analysis of scientific literature [44].

*HDABCE1* was identified in *H. doenitzi* and is part of the ABC transporter superfamily. These proteins are common in eukaryotes and use ATP energy to transport substances across membranes against a concentration gradient [45]. ABCEs help regulate gene expression, immune responses, and detoxification to keep cells stable [46]. They also reduce exposure to toxins, including pesticides, playing a key role in cellular and organismal toxicity [47]. The *HDABCE1* gene expression was higher in *O. onites* EO than in controls but lower in carvacrol. In *O. onites* EO-treated adults, increased Na^+^/K^+^-ATPase expression helps maintain the Na^+^ and K^+^ balance, which is vital for physiological processes like action potentials [48]. Carvacrol and *O. onites* EO decreased the expression of ABC transporter genes, matching earlier findings [49]. The results of enzyme activity assays showed that *O. onites* EO and carvacrol were able to inhibit ATPase activity, which may interfere with the ionic homeostasis of the cell, thus affecting the physiological function of the cell and the stress response [50]. Cinnamon EO affects energy metabolism by inhibiting adenosine triphosphate (ATP) levels and ATPase activity [51]. In insects, silencing the *ABCC2* gene resulted in increased larval mortality [52]. These findings suggest that inhibition of ATPase activity is important in tick toxicology studies and can be a potential target for the development of novel acaricides.

ABC transporters and GST collaborate in the detoxification process of arthropods. GST increases the water solubility of exogenous compounds during phase II metabolism, while ABC transporters expel these products from the cell in phase III [53]. GST is a common enzyme in aerobic organisms, involved in detoxification, intracellular transport, hormone synthesis, and anti-oxidative stress [54]. It was found that the expression of *HD-GSTa* gene was significantly increased after treatment with the LC_50_ *O. onites* EO and carvacrol. *Haemaphysalis longicornis* treated with LC_50_ cyhalothrin was also found to have significantly increased expression of the *HrGSTm1* gene in the tick’s midgut, ovaries, salivary glands, and Malpighian tubule [55]. *Origanum onites* EO was found to significantly inhibit the GST enzyme activity of *H. doenitzi*, while carvacrol had no significant effect on the tick. The same trend was observed for GST enzyme activity in oregano EO-treated *Agrotis ipsilon* [56]. GST is critical for tick resistance to acaricides, aiding in detoxification and reducing intracellular toxin accumulation, and investigating its mechanisms can help improve the efficiency of plant resource utilization in tick control.

In addition to GST and ABC transporters, CYP450 is also key in tick detoxification [57]. CYP450, a phase I detoxification enzyme, is the first line of defense against *O. onites* EO. These enzymes make exogenous substances more polar through oxidation and other reactions, which aids in their excretion [58]. The expression of the *HD-CYP450a* gene was significantly increased and decreased after treatment with LC_50_ *O. onites* EO and carvacrol, respectively. This may indicate that *O. onites* EO has a complex composition and that there may be antagonism between carvacrol and other components [59]. Previous studies demonstrated insecticide-mediated upregulation of the CYP450 gene in *Tribolium castaneum* [60]. CYP450 *CYP9A14* and *CYP6AE11* transcripts were significantly upregulated in *Helicoverpa armigera* following different insecticide treatments [61]. CYP450 is important in the acaricidal mechanism of plant EOs, and its complex interactions with EO components provide new ideas for tick control and prevention, helping to optimize formulations and improve the efficiency of plant resource use [62]. In addition, AchE activity was found to be significantly inhibited after treatment with LC_50_ *O. onites* EO and carvacrol. Carvacrol significantly inhibits AChE activity, interferes with neurotransmission, and exerts acaricidal effects in *H. longicornis* [63]. It was found that CarE activity was significantly increased after *O. onites* EO treatment, suggesting that CarE enhances detoxification efficacy through enhanced drug metabolism [64]. These results suggest that *O. onites* EO and carvacrol may regulate the detoxification mechanism of ticks by affecting the activity of these key enzymes, thereby influencing their tolerance to toxins.

The molecular docking results validated ABC transporters, GST, and CYP450 as potential targets, and also revealed subtle differences in the interactions between the two monomers and the protein. Carvacrol molecules lack the nitrogen and oxygen heteroatoms necessary for forming hydrogen bonds. Consequently, they primarily rely on hydrophobic stacking and van der Waals forces to maintain binding within the active pockets of ABC transporters and GST [65]. These molecules are unable to form classical hydrogen bonds. This is consistent with the observed phenomenon of GST gene upregulation without changes in enzyme activity, suggesting that transcriptional induction precedes functional compensation, and is also consistent with reports in *H. longicornis* [63]. However, carvacrol has the lowest binding free energy (−3.65 kcal/mol) for ABC transporters, indicating its strongest overall affinity; at the same time, it can form three hydrogen bonds with Ser-90 within the substrate channel of CYP450. In comparison, although eugenol possesses hydroxyl hydrogen bonding ability, because of its large molecular size and insufficient hydrophobic surface area, its binding energy is higher than that of carvacrol on all three proteins, indicating overall weaker affinity. These differences explain the high efficacy of carvacrol and provide a basis for the design of targeted plant-based acaricides. Carvacrol as an insecticide has been shown in several studies to exert its effects by inhibiting AchE activity and disrupting the integrity of cell membranes [66, 67]. In addition, carvacrol has been widely investigated for use in food preservation, antimicrobial, antiparasitic, and anti-inflammatory applications, demonstrating its multifunctional application potential [68, 69]. These results suggest that *O. onites* and *O. gratissimum* EOs have great potential for practical application as plant-derived pesticides for pest control. A limitation of this study is that other components in EOs may also play a role in the acaricidal effect; thus, their specific mechanisms and contributions require further investigation.

## Conclusions

In this study, *O. onites* EO, *O. gratissimum* EO, and carvacrol showed high acaricidal activity against *H. doenitzi*. *Origanum onites* EO and DEET showed high repellent activity against ticks. ABC transporter, GST, and CYP450 are involved in the toxin mechanism of *H. doenitzi*. Molecular docking studies further verified that carvacrol and eugenol regulate these three proteins for tick control. These findings provide a framework for elucidating the biochemical defense network of the bloodsucking tick and inform the development of pest management strategies focused on detoxification and metabolism-related enzymes. Future studies could explore the nano-encapsulation or microemulsion of carvacrol for extended hold, reduced toxicity, and semi-field validation in naturally tick-infested livestock.

## Supplementary Information

Supplementary material 1.

## Data Availability

Data supporting the main conclusions of this study are included in the manuscript.
