# Supplementary material for: Acaricidal bioactivity and molecular target analysis of Origanum onites and Ocimum gratissimum essential oils against Haemaphysalis doenitzi ticks
Source: Parasit Vectors. 2025 Oct 8;18:401. doi: 10.1186/s13071-025-07031-3 (PMC12506390; doi:10.1186/s13071-025-07031-3)
Supplement: Supplementary file 1 — Supplementary material 1. [file 13071_2025_7031_MOESM1_ESM.docx]

Table S1 Primers for polymerase chain reaction (PCR)

| Method | Gene | Forward primer (5′→3) | Reverse primer (5′→3) |
| --- | --- | --- | --- |
| Quantitative real-time PCR | *HD-ABCE1* (PQ877926) | GGCTTCGTGCCAACTGAGA | TGTCTTCCCCGTGCCGTT |
|  | *HD-CYP450a* (PP962429) | GCATGGGTCCTCGAAACTG | GCCGGAACAGCGAAGATAG |
|  | *HD-GSTa* (PQ657472) | GGCCGTGGAGCTGTACAA | TGATGGTCGGCACCGTAT |
|  | *β-Actin* (AY254898) | CGTTCCTGGGTATGGAATCG | TCCACGTCGCACTTCATGAT |
